# Association of Sleep Quality, Sleep Disturbances, and Chronotype with Post-Traumatic Stress Disorder in Earthquake-Exposed Adolescents

**DOI:** 10.3390/children13030423

**Published:** 2026-03-19

**Authors:** Gürkan Temelli, Yunus Emre Dönmez

**Affiliations:** Department of Child and Adolescent Psychiatry, Inonu University, Malatya 44280, Türkiye; gurkantemelli@gmail.com

**Keywords:** post-traumatic stress disorder, adolescence, sleep quality, chronotype, sleep disturbance, earthquake

## Abstract

**Highlights:**

**What are the main findings?**

- Adolescents with PTSD after the 2023 Türkiye earthquakes exhibited poorer sleep quality and more pronounced sleep problems than earthquake-exposed controls.
- Sleep quality impairment and clinically significant sleep problems were independently associated with PTSD status and correlated with symptom severity.
- Evening chronotype was more frequent in adolescents with PTSD and associated with symptom severity but was not independently linked to PTSD in multivariable analyses.

**What are the implications of the main findings?**

- Routine assessment of sleep disturbances may help identify adolescents at risk for persistent PTSD symptoms after disasters.
- Interventions targeting sleep, including cognitive behavioral therapy for insomnia and nightmare-focused treatments, may improve sleep outcomes and reduce PTSD symptom severity.

**Abstract:**

**Background/Objectives:** Post-traumatic stress disorder (PTSD) is a common psychiatric consequence of trauma, and adolescents may be particularly vulnerable after large-scale disasters. Sleep disturbances and circadian preference may play a role in PTSD symptomatology. This study aimed to investigate the associations between PTSD, sleep quality, sleep problems, and chronotype in adolescents exposed to an earthquake. **Methods:** This cross-sectional study included 201 adolescents aged 12–18 years: 92 diagnosed with PTSD and 109 earthquake-exposed controls without a DSM-5 psychiatric disorder. Participants completed the Children’s Posttraumatic Stress Reaction Index (CPTS-RI), Pittsburgh Sleep Quality Index (PSQI), Children’s Sleep Habits Questionnaire (CSHQ), and Children’s Chronotype Questionnaire (CCTQ). Group differences, correlation analyses, and binary logistic regression analyses were performed. **Results:** Adolescents with PTSD had significantly higher CPTS-RI, PSQI, CSHQ, and CCTQ scores compared with controls (all *p* < 0.001), indicating poorer sleep quality, more sleep problems, and a greater tendency toward eveningness. PTSD severity was positively correlated with sleep problems, impaired sleep quality, and eveningness. In logistic regression analysis, poor sleep quality (*p* < 0.001) and clinically significant sleep problems (*p* = 0.011) were independently associated with PTSD, whereas chronotype was not. **Conclusions:** Sleep disturbances are more strongly associated with PTSD than chronotype in earthquake-exposed adolescents. Assessment and treatment of sleep problems may represent an important therapeutic target following large-scale trauma.

## 1. Introduction

Exposure to or witnessing events such as war, accidents, natural disasters, or sexual assault can elicit a stress response in most individuals; however, in some, these experiences lead to the development of post-traumatic stress disorder (PTSD). PTSD is one of the most common psychiatric conditions following traumatic exposure and is characterized by four core symptom clusters: re-experiencing, avoidance, negative alterations in cognition and mood, and hyperarousal [1]. From a neurobiological perspective, PTSD is associated with dysregulation in multiple stress-response systems, including heightened amygdala reactivity, impaired top-down regulation by the prefrontal cortex, and alterations in hypothalamic–pituitary–adrenal (HPA) axis functioning. These neurobiological alterations are believed to contribute to persistent hyperarousal, intrusive recollections, and impaired emotional regulation following trauma.

Both individual and trauma-related factors influence the likelihood of developing PTSD. Individual factors include sex, previous traumatic experiences, and a history of psychiatric disorders, whereas trauma-related characteristics such as trauma severity, perceived threat to life, and physical injury also play a critical role [2]. Children and adolescents represent particularly vulnerable populations, as they have an increased risk of both trauma exposure and subsequent PTSD compared with adults [3]. Developmental neurobiology may partially explain this increased vulnerability. During adolescence, neural circuits involved in emotion regulation, threat detection, and cognitive control are still maturing. This ongoing neurodevelopment may reduce resilience to traumatic stress and increase susceptibility to trauma-related psychopathology [4].

Although the cognitive, emotional, behavioral, and somatic manifestations of PTSD in adolescents are broadly similar to those observed in adults, the disorder may have particularly profound consequences for psychosocial functioning, academic achievement, and emotional development [5]. The prevalence of PTSD among trauma-exposed children and adolescents has been estimated at approximately 15.9%, with even higher rates reported following large-scale traumatic events such as natural disasters, armed conflicts, and mass violence [6]. Understanding the mechanisms underlying PTSD in adolescents is therefore especially important, as symptom expression, neurodevelopmental vulnerability, and recovery trajectories may differ substantially from those observed in adults [7]. A better understanding of these mechanisms may also inform treatment strategies, which currently include evidence-based psychological interventions—particularly trauma-focused cognitive behavioral therapy—as well as pharmacological approaches targeting specific symptom domains.

Increasing evidence suggests that sleep disturbances and circadian rhythm dysregulation play a central role in the pathophysiology and clinical manifestation of PTSD. Sleep is critically involved in emotional memory consolidation, fear extinction, and neural network recalibration. Consequently, sleep disturbances may disrupt adaptive processing of traumatic experiences and contribute to the persistence of PTSD symptoms. Prospective studies indicate that sleep disturbances present prior to trauma exposure may increase vulnerability to trauma-related psychiatric disorders [8]. From a mechanistic perspective, sleep contributes to the restoration and recalibration of neuronal networks essential for emotional and cognitive functioning [9]. Sleep disruption may impair cognitive performance, worsen mood regulation, and produce daytime fatigue, thereby amplifying psychological distress and functional impairment in individuals with PTSD [10].

Sleep regulation involves the interaction between sleep–wake homeostasis and the circadian timing system. In PTSD, persistent hyperarousal and stress-related alterations in circadian phase may disrupt the sleep–wake cycle and alter sleep architecture [11]. According to the hyperarousal model of insomnia, sustained physiological and cognitive activation following trauma may interfere with both sleep initiation and sleep maintenance, contributing to insomnia symptoms frequently observed in individuals with PTSD [12]. Importantly, the relationship between sleep disturbances and PTSD appears to be bidirectional: trauma-related hyperarousal may disrupt sleep, while persistent sleep disturbances may in turn exacerbate emotional dysregulation, impair fear extinction, and maintain PTSD symptomatology.

Sleep disturbances are explicitly included among the DSM-5 diagnostic criteria for PTSD and represent one of the most frequent clinical complaints among affected individuals [1]. PTSD has been associated with a wide range of sleep-related problems, including insomnia, nightmares, periodic limb movements, sleep apnea, excessive daytime sleepiness, and sleep-related anxiety, with some studies reporting comorbidity rates approaching 90% [13]. Sleep disturbances may impair fear extinction processes and thereby contribute to the persistence of conditioned fear responses following trauma. Furthermore, several studies have demonstrated that the severity of sleep disturbances is positively correlated with the severity of PTSD symptoms [14].

Although sleep disturbances are widely recognized as a core feature of PTSD, emerging evidence suggests that the circadian system may also influence both sleep regulation and vulnerability to trauma-related psychopathology [15]. Circadian rhythms are approximately 24 h biological cycles generated by endogenous biological clocks and synchronized by environmental cues known as zeitgebers. These rhythms regulate numerous physiological and behavioral processes, including sleep–wake patterns, hormone secretion, body temperature, and cognitive functioning. Chronotype reflects individual differences in circadian preference and represents a behavioral manifestation of circadian timing mechanisms. Thus, chronotype reflects both biological circadian regulation and behavioral or psychological preferences regarding the timing of daily activities [16,17].

Three chronotype categories are typically described: morning, intermediate, and evening types. Morning types tend to fall asleep and wake earlier and demonstrate optimal cognitive and physical performance during the early hours of the day. Evening types typically go to bed later, wake later, and perform better during the evening hours, whereas individuals without a clear preference are classified as intermediate types [16]. Chronotype changes across the lifespan, and a shift toward eveningness is particularly pronounced during adolescence [18]. This developmental shift may increase the likelihood of circadian misalignment between adolescents’ biological rhythms and socially imposed schedules, such as early school start times.

Circadian rhythm disruption has been associated with various psychiatric disorders, neurodevelopmental conditions, and medical illnesses. Misalignment between biological circadian timing and daily social demands is particularly common among evening types and may contribute to circadian disruption [19]. Disrupted circadian rhythms may reduce both sleep quantity and sleep quality, resulting in social jet lag and chronic stress [20]. Additionally, evening chronotype has been linked to emotional dysregulation, reduced behavioral activation, and increased vulnerability to mental health problems [21]. Consistent with these findings, studies conducted in both adults and adolescents have reported higher rates of mood disorders, sleep disturbances, and substance use disorders among individuals with an evening chronotype [20,22].

In adult populations, several studies examining the relationship between PTSD and circadian characteristics have suggested that evening chronotype may represent a potential risk factor [23,24,25,26]. However, findings remain inconsistent, as other studies have failed to demonstrate a significant association between PTSD and evening chronotype [15,27]. In children and adolescents, research examining the relationship between PTSD, circadian rhythm disturbances, and chronotype remains limited. In a study involving 68 children and adolescents aged 8–17 years following Hurricane Harvey, evening chronotype and self-reported sleep problems were associated with post-traumatic stress symptoms. Participants with an evening chronotype also demonstrated higher levels of re-experiencing, hyperarousal, and cognitive distortions, while shorter sleep duration measured via actigraphy was associated with avoidance symptoms. The authors suggested that post-disaster hyperarousal combined with evening chronotype may contribute to sleep disturbances, which in turn may impair emotional regulation and increase post-traumatic stress symptoms [28]. Similarly, another small study comparing PTSD cases aged 3–18 years with controls reported that sleep disturbances and evening chronotype characteristics were associated with PTSD [29]. Nevertheless, it remains unclear whether chronotype is independently associated with PTSD or whether the observed relationship may primarily be explained by underlying sleep disturbances.

Importantly, large-scale natural disasters such as earthquakes represent particularly powerful traumatic events that may disrupt both psychological functioning and biological rhythms. Adolescents exposed to such disasters may experience persistent environmental stressors, including aftershocks, displacement, and changes in daily routines, which may further disrupt sleep patterns and circadian regulation. Despite these considerations, relatively few studies have examined the combined roles of sleep disturbances and chronotype in adolescents exposed to large-scale disasters.

Considering the available literature, studies examining the association between PTSD and chronotype in adolescents remain limited. Based on previous findings, we hypothesized that adolescents with PTSD would exhibit poorer sleep quality, more sleep problems, and a greater tendency toward evening chronotype compared with earthquake-exposed controls. Furthermore, we hypothesized that sleep disturbances would independently predict PTSD status beyond chronotype characteristics. This study aimed to provide further insight into the relationship between sleep disturbances, sleep quality, and chronotype in adolescents with PTSD following earthquake exposure.

## 2. Materials and Methods

This cross-sectional case–control study examined the relationships between PTSD, sleep quality, sleep problems, and chronotype in adolescents exposed to an earthquake.

### 2.1. Participants

On 6 February 2023, the Kahramanmaras Earthquake Sequence (Mw 7.8 and Mw 7.5) occurred in southern Türkiye and caused major destruction across eleven provinces. This disaster sequence affected more than 9.1 million people and resulted in more than 51,000 deaths [30]. The present study was conducted among adolescents who presented to the İnönü University Faculty of Medicine Department of Child and Adolescent Psychiatry outpatient clinic following the Kahramanmaras Earthquake Sequence. The study protocol was approved by the İnönü University Clinical Research Ethics Committee (2023/70) and conducted in accordance with the Declaration of Helsinki.

Earthquake-exposed adolescents aged 12–18 years who presented to the outpatient clinic for psychiatric evaluation were considered for inclusion. Participants and their parents or legal guardians were informed about the study procedures, and written informed consent was obtained before enrollment.

#### 2.1.1. Inclusion Criteria

Participants were included if they:

(a) were between 12 and 18 years of age,
(b) had been directly exposed to the Kahramanmaras Earthquake Sequence,
(c) presented to the child and adolescent psychiatry outpatient clinic for psychiatric evaluation, and
(d) provided written informed consent from both the adolescent and at least one parent/legal guardian.

#### 2.1.2. Exclusion Criteria

Participants were excluded if they had:

(a) autism spectrum disorder,
(b) psychotic disorder,
(c) bipolar disorder,
(d) any psychiatric disorder known to affect circadian rhythms, or
(e) any chronic medical illness.

### 2.2. Procedure

After obtaining ethical approval, adolescents aged 12–18 years who were exposed to the Kahramanmaras Earthquake Sequence and presented to the İnönü University Faculty of Medicine Department of Child and Adolescent Psychiatry outpatient clinic were consecutively evaluated. Data collection was conducted between December 2023 and June 2024, approximately 10–16 months after the Kahramanmaras Earthquake Sequence.

Psychiatric assessments were conducted through face-to-face clinical interviews performed by experienced child and adolescent psychiatrists with at least five years of clinical practice, according to DSM-5 diagnostic criteria. Based on these evaluations, participants were assigned either to the PTSD group (meeting full DSM-5 diagnostic criteria for PTSD) or to an earthquake-exposed control group whose members underwent the same clinical evaluation and were confirmed to have no current DSM-5 psychiatric disorder.

Following the clinical evaluation, adolescents completed self-report questionnaires assessing post-traumatic stress symptoms and sleep quality. Parents or primary caregivers completed parent-report questionnaires assessing sleep habits and chronotype. All questionnaires were administered during the same clinical visit under the supervision of the research team to ensure completeness of the data.

Participation in this study was voluntary. Both adolescents and their parents or legal guardians received detailed information about the study procedures.

### 2.3. Measures

#### 2.3.1. Sociodemographic Questionnaire

The sociodemographic questionnaire was developed by the researchers and included questions to obtain demographic information (age, sex, education level, family structure, and economic status), as well as earthquake-related questions.

#### 2.3.2. Children’s Posttraumatic Stress Reaction Index (CPTS-RI)

The CPTS-RI is a 20-item, 5-point Likert-type self-report measure used to assess PTSD symptoms that develop after a traumatic event. Each item is scored from 0 to 4, and Items 7, 12, and 15 are reverse-coded. Based on total scores, symptoms are classified as mild (12–24), moderate (25–39), severe (40–59), or very severe (≥60) [31]. The validity and reliability of the Turkish version were evaluated by Erden and colleagues in 1999 [32]. In that study, test–retest reliability was 0.86, Cronbach’s alpha was 0.75, and inter-rater agreement (kappa) was 0.887. All items refer to a specific traumatic event; in the present study, the traumatic event was the Kahramanmaras Earthquake Sequence (Mw 7.8 and Mw 7.5) that occurred in southern Türkiye on 6 February 2023.

#### 2.3.3. Children’s Sleep Habits Questionnaire (CSHQ)

The CSHQ is a parent-reported questionnaire developed by Owens et al. to investigate sleep habits and sleep-related problems in school-aged children [33]. The scale demonstrated adequate internal consistency in both community and clinical samples. The 33-item questionnaire is typically coded as usually (behavior occurs 5–7 times per week) = 3, sometimes (2–4 times per week) = 2, or rarely (0–1 time per week) = 1; items 1, 2, 3, 10, 11, and 26 are reverse-coded (usually = 1, sometimes = 2, rarely = 3). A total score of 41 is commonly used as a cutoff, with values above this threshold interpreted as clinically significant sleep problems. The Turkish validity and reliability of the short form were evaluated by Fiş and colleagues, with test–retest reliability of 0.81 and Cronbach’s alpha of 0.78 [34].

#### 2.3.4. Pittsburgh Sleep Quality Index (PSQI)

The PSQI is a measure developed by Buysse et al. to assess sleep quality based on sleep habits, sleep problems, and their impact on daytime functioning [35]. It is a 19-item self-report scale assessing subjective sleep quality over the past month, with a total score ranging from 0 to 21; scores > 5 are associated with poor sleep quality. The Turkish validity and reliability study was conducted by Ağargün and colleagues [36].

#### 2.3.5. Children’s Chronotype Questionnaire (CCTQ)

The CCTQ is a 27-item parent-report questionnaire comprising 16 items assessing sleep–wake parameters on scheduled days and free days, a 10-item morningness–eveningness scale, and a final item evaluating chronotype. Items 17–26 are rated on a 5-point scale (1–5). In the final item (Item 27), parents classify their child’s chronotype by selecting one of the following options: “Definitely a Morning Type,” “Rather a Morning Type than an Evening Type,” “Neither a Morning nor an Evening Type,” “Rather an Evening Type than a Morning Type,” “Definitely an Evening Type,” or “I do not know.” Items 17, 18, 24, and 25 are reverse-scored, yielding a total morningness–eveningness score ranging from 10 to 48; scores ≤ 23 indicate morning type, 24–32 intermediate type, and ≥33 evening type [37]. The Turkish validity and reliability study was conducted by Dursun et al., who reported a Cronbach’s alpha of 0.65 [38].

### 2.4. Statistical Analysis

An a priori power analysis was conducted using G*Power 3.1. Assuming a medium effect size (d = 0.5), α = 0.05, and power (1 − β) = 0.80, the minimum required sample size was 128 participants. The final sample size of 201 participants exceeded this requirement.

Data analysis was performed using SPSS version 22.0. Descriptive statistics were presented as mean ± standard deviation, number, and percentage. Data distribution was assessed using the Shapiro–Wilk test. For parametric data, the independent-samples *t*-test was used, and Pearson’s chi-square test or Fisher’s exact test was applied for categorical variables, as appropriate. Associations between variables were evaluated using Pearson correlation analysis. Multicollinearity was assessed using variance inflation factor (VIF) values before regression analyses, and no significant multicollinearity was detected. The relationship between PTSD and sleep quality, sleep problems, and chronotype was tested using binary logistic regression analysis. A *p*-value < 0.05 was considered statistically significant.

## 3. Results

A total of 201 adolescents participated in this study: 92 in the PTSD group and 109 in the control group. The mean age was 15.30 ± 2.04 years in the PTSD group and 14.80 ± 1.87 years in the control group. The PTSD group included 60 girls (65.2%) and 32 boys (34.8%), whereas the control group included 60 girls (55.0%) and 49 boys (45.0%).

There were no significant between-group differences in age, sex, place of residence, income level, parental psychiatric history, earthquake-related home damage, or earthquake-related bereavement. When post-earthquake accommodation was considered, the PTSD group reported a lower rate of out-of-province accommodation compared with the control group (*p* = 0.047). Sociodemographic and earthquake-related data are presented in Table 1.

There were statistically significant between-group differences in CPTS-RI (post-traumatic stress severity), PSQI (sleep quality), CSHQ (sleep problems), and CCTQ (chronotype) scores (all *p* < 0.001). Compared with the control group, the PTSD group had higher mean CPTS-RI, PSQI, CSHQ, and CCTQ scores. Scale score data are presented in Table 2.

Based on CPTS-RI scores in the PTSD group, 74 participants (80.4%) had severe symptoms and 18 (19.6%) had very severe symptoms. Symptom severity categories differed between groups (*p* < 0.001). Regarding chronotype, the intermediate type was more common in the control group, whereas the evening type was more prevalent in the PTSD group (*p* < 0.001).

There were marked differences between the PTSD and control groups in sleep quality and sleep problems. Adolescents with PTSD reported poorer sleep quality and more sleep problems (*p* < 0.001). Restlessness during sleep and nightmares were more frequent in the PTSD group (*p* < 0.001 and *p* = 0.005, respectively). Analyses of CPTS-RI-, CCTQ-, PSQI-, and CSHQ-related categorical data are presented in Table 3.

Pearson correlation analyses showed that CPTS-RI, PSQI, CSHQ, and CCTQ scores were positively correlated with one another (all *p* < 0.001), indicating that greater PTSD symptom severity was associated with poorer sleep quality, more sleep problems, and a greater tendency toward eveningness. Correlation data are presented in Table 4.

In binary logistic regression analyses, poor sleep quality and clinically significant sleep problems were independently associated with PTSD status (*p* < 0.001 and *p* = 0.017, respectively). Although chronotype differed between groups, chronotype characteristics were not independently associated with PTSD status in the multivariable model.

To further examine the robustness of the findings, a sensitivity analysis was conducted by adding out-of-province accommodation—the only sociodemographic variable that differed significantly between groups—into the logistic regression model. After adjustment for this variable, poor sleep quality (OR = 9.378) and clinically significant sleep problems (OR = 2.716) remained independently associated with PTSD status.

In an additional sensitivity analysis using the continuous CCTQ total morningness–eveningness score instead of categorical chronotype groups, the CCTQ score was not independently associated with PTSD status (*p* > 0.05), and the overall pattern of results remained unchanged. Logistic regression results are presented in Table 5.

## 4. Discussion

The present findings indicate that sleep disturbances are a prominent feature of adolescent PTSD following disaster exposure. Both impaired sleep quality and increased sleep problems were independently associated with PTSD and correlated with symptom severity. Although evening chronotype was more frequent among adolescents with PTSD and associated with greater symptom severity, chronotype was not independently associated with PTSD in regression analyses. Taken together, these findings suggest that sleep disturbances, rather than chronotype itself, may be more closely associated with PTSD among adolescents.

Poor sleep quality and sleep problems co-occur with many psychiatric disorders and are considered core features of PTSD [13,39]. In PTSD, sleep quality may deteriorate due to hyperarousal, increased sleep disturbances, sleep-related anxiety, and disrupted circadian rhythms [40]. This may contribute to the persistence of PTSD symptoms and functional impairment, and a positive correlation has been reported between PTSD symptom severity and impaired sleep quality [41]. Studies in adolescents, similar to those in adults, show that trauma exposure is associated with poorer sleep quality [42,43]. Adolescents who developed PTSD after sexual assault have been reported to have poorer sleep quality than controls, and sleep problems were reported to affect quality of life more strongly than PTSD itself [44]. Moreover, sleep impairment may persist even after clinical improvement with psychotherapy or other treatments, suggesting that poor sleep quality and sleep problems can continue beyond other PTSD symptoms [45].

Individuals with PTSD frequently report reduced sleep quality, frequent awakenings, insomnia, nightmares, excessive daytime sleepiness, and impaired functioning [46]. Increased amygdala activity and dysregulated neuroendocrine systems after trauma have been proposed to contribute to insomnia via hyperarousal. High anxiety, rumination, and cognitive distortions may further increase hyperarousal and disrupt sleep quality [47]. Sleep is critical for emotional memory consolidation and stress regulation. While sleep disruption may impair effective fear extinction and has been linked to the development of intrusive thoughts and nightmares in PTSD [48], it is important to recognize that this relationship is likely bidirectional. Core PTSD symptoms, such as hyperarousal and re-experiencing, act as significant physiological and psychological stressors that disrupt sleep. This may create a reciprocal cycle in which poor sleep compromises emotional resilience, and ongoing PTSD symptoms further degrade sleep quality. For instance, PTSD-related nightmares can trigger sleep-related anxiety, which may lead to a persistent pattern of sleep avoidance and maintenance insomnia [49]. Insomnia and nightmare disorder are commonly reported after trauma and have also been described in studies of adolescents following earthquakes [43,50,51]. In the present study, the PTSD group had markedly poorer sleep quality and significantly more sleep problems than the control group. Correlation and binary logistic regression analyses indicated that PTSD symptom severity was associated with impaired sleep quality and sleep problems, and that poor sleep quality and clinically significant sleep problems were independently associated with PTSD status.

In addition to statistical significance, the magnitude of the observed associations appears clinically meaningful. Logistic regression indicated that adolescents with impaired sleep quality had approximately nine times higher odds of PTSD, suggesting a strong association between sleep quality and PTSD status. Clinically significant sleep problems were also associated with nearly threefold higher odds of PTSD, indicating a moderate but meaningful effect. Consistent with these findings, group comparisons demonstrated large standardized effect sizes, particularly for sleep quality (Cohen’s d = 1.42) and sleep problems (Cohen’s d = 0.97), while chronotype showed a moderate effect size (Cohen’s d = 0.64). These findings highlight the potential clinical importance of assessing sleep disturbances in adolescents exposed to large-scale traumatic events. Systematic evaluation of sleep disturbances may represent an important component of early identification and intervention strategies for PTSD in disaster-exposed adolescents.

Sleep-focused psychological interventions may represent promising approaches for trauma-related sleep disturbances. Cognitive behavioral therapy for insomnia (CBT-I) has been shown to improve sleep outcomes in adolescents, including sleep onset latency, total sleep time, and sleep efficiency [52]. In addition, nightmare-focused interventions such as imagery rehearsal therapy have demonstrated beneficial effects in reducing nightmare frequency and improving sleep quality in trauma-related sleep disturbances [53]. However, most intervention studies have been conducted in adults or in adolescents with primary insomnia, and evidence specifically focusing on disaster-exposed adolescents remains limited. Future research should evaluate whether sleep-focused interventions may help reduce PTSD symptoms in disaster-exposed adolescents.

Many studies have reported that chronotype may be associated with certain psychiatric problems, and eveningness has been linked to mood disorders, sleep disorders, attention-deficit/hyperactivity disorder, and substance use [54]. However, evidence regarding the relationship between evening chronotype and PTSD is derived largely from adult populations, and findings remain limited and inconsistent. Some studies examining the link between chronotype and PTSD have reported an association with eveningness. In a 2023 study including 120 adults and using the Trauma and Loss Spectrum–Self Report (TALS), PSQI, a short MEQ, and actigraphy, eveningness was associated with poorer sleep quality and higher TALS scores [55]. A twin study examined the relationship between PTSD and chronotype from a genetic perspective using the Impact of Events Scale (IES) and a short MEQ; it reported a significant association between eveningness and PTSD symptom severity in adults and suggested a partially causal genetic relationship [56]. Hasler et al. also reported findings indicating that eveningness may be associated with poorer sleep quality and PTSD in adults [23]. In a study using the PTSD Checklist for DSM-5 (PCL-5), Dissociative Experiences Scale, PSQI, and MEQ, the dissociative PTSD group exhibited a greater tendency toward eveningness [57]. Another study investigated the impact of chronotype on PTSD among medical students after the 6 February Kahramanmaras Earthquakes; after administration of the MEQ and PCL-5, participants with an evening chronotype reported higher PCL-5 scores than other types, and eveningness was discussed as a potential risk factor for PTSD [26]. However, other studies have reported no association between PTSD and eveningness [15,27]. In the meta-analysis by Zalta et al. examining sleep timing, chronotype, and PTSD, only two adult studies assessing the chronotype–PTSD relationship were included, and no clear association was identified [15]. In the present study, although correlation analyses suggested a possible association between eveningness and PTSD, binary logistic regression indicated that chronotype was not independently associated with PTSD status. However, given the cross-sectional design, it remains unclear whether eveningness contributes to PTSD symptoms, whether PTSD influences circadian preference, or whether shared underlying mechanisms explain this association. Although formal mediation analyses require longitudinal data, the pattern observed in our results is consistent with the possibility that sleep disturbances may partly explain the association between chronotype and PTSD. This interpretation is consistent with previous research suggesting that sleep disturbances may represent an important pathway linking circadian preference and PTSD-related symptoms, particularly through mechanisms involving hyperarousal and disrupted sleep regulation [39].

One possible explanation may relate to measurement-related factors. In the present study, chronotype was assessed using a parent-report measure, whereas sleep quality was based on adolescent self-report. Differences in informant source may contribute to measurement variability and could partly explain why chronotype was not independently associated with PTSD in multivariable analyses.

In the literature, the relationship between chronotype and PTSD is often discussed in the context of sleep problems [23,25]. For example, studies have suggested that poorer sleep quality may be associated with greater post-traumatic stress symptoms, whereas chronotype may not remain independently associated after accounting for sleep-related variables [55,58]. Our results appear largely consistent with these findings.

Taken together, our findings support the hypothesis that the relationship between chronotype and PTSD may be partly explained by sleep disturbances. Eveningness may be associated with poorer sleep quality and increased sleep problems, which in turn are linked to PTSD symptoms. Therefore, chronotype itself may not constitute an independent correlate of PTSD but rather an indirect marker operating through sleep-related mechanisms.

These findings may have clinical implications. Assessment of sleep disturbances should be considered a routine component of the evaluation of adolescents with PTSD after disasters. Interventions targeting sleep problems, such as sleep hygiene education, cognitive behavioral therapy for insomnia, and nightmare-focused interventions, may contribute not only to improved sleep but also to reduced PTSD symptom severity. Therefore, sleep disturbances may represent a modifiable treatment target in adolescent PTSD.

Beyond trauma characteristics, socioeconomic and family factors also play an important role in PTSD development. Low income, disadvantaged environmental conditions, and parental psychiatric disorders have been described as PTSD risk factors [59]. Significant associations have been reported between parental psychological functioning and children’s PTSD symptoms [60]. In the present study, groups were similar with respect to place of residence, household income, and parental psychiatric disorder. In addition, the PTSD and control groups were comparable with respect to trauma-related variables, including earthquake-related home damage and bereavement. Therefore, the association observed between sleep disturbances and PTSD is unlikely to be explained by differences in trauma exposure or socioeconomic background.

As noted in the literature, female sex, living in rural areas, low household income, and parental psychiatric disorders are considered risk factors for PTSD. The primary aim of the present study was to examine the relationship between sleep problems, sleep quality, and chronotype in adolescents with PTSD, and it was not designed as an epidemiological study. In this context, between-group similarity in key sociodemographic variables that could act as confounders (age, sex, place of residence, income, and parental psychiatric disorder) increases the credibility of the findings related to the study aims.

Several studies have reported that injury or bereavement after disasters may increase PTSD risk [61], and similar results were reported after the Kahramanmaras Earthquake Sequence [62,63]. In addition, material losses and earthquake-related home damage, which may reflect trauma severity, have also been reported as potential PTSD risk factors [64]. In the present study, there were no significant between-group differences in bereavement or home damage. In addition, trauma-related variables such as earthquake-related home damage and bereavement were comparable between the PTSD and control groups, suggesting that the observed associations between sleep disturbances and PTSD were unlikely to be explained by differences in trauma severity. This may be related to the small number of participants who experienced bereavement. Moreover, given that much of the available literature focuses on adult populations, direct comparison may not be appropriate. Although trauma-related variables such as home damage, bereavement, and relocation were examined descriptively, the present study was not designed to test moderation effects between trauma severity and sleep-related variables. Future studies with larger samples and longitudinal designs may help clarify whether trauma severity moderates the relationship between sleep disturbances and PTSD symptoms.

Adverse experiences and repeated traumas in the aftermath of an earthquake are associated with a higher risk of PTSD [61]. In the present study, residing in provinces not affected by the earthquake after the disaster was significantly more common in the control group than in the PTSD group. This pattern may reflect reduced exposure to trauma reminders, which have been linked to an increased risk of PTSD. Consistent with this interpretation, traumatic events occurring before or after an index trauma are associated with higher PTSD prevalence in adolescents [65]. Aftershocks persisted for a year following the Kahramanmaras Earthquake Sequence, and ongoing residence in the epicenter or affected regions may involve recurrent exposure to smaller earthquakes and persistent reminders of destruction, which may disrupt normalization and recovery processes and limit access to effective psychosocial support, potentially contributing to PTSD.

Studies suggest that the highest risk of trauma exposure occurs between ages 16 and 20 years [66]. However, a 2024 systematic review and meta-analysis examining PTSD development after traumatic injuries suggested that age may be only a weak predictor [67]. In the meta-analysis by Tang et al., including earthquake-exposed children and adults, age was not reported as a PTSD risk factor after correction for publication bias [61]. In the present study, consistent with the findings of Tang et al., there was no significant age difference between the groups. Nevertheless, because only adolescents aged 12–18 years were evaluated and the study design is cross-sectional, it is not appropriate to make broad generalizations about the relationship between age and PTSD.

In the literature, the distribution of PTSD by sex is approximately 2:1 in favor of females. Studies of earthquake survivors have consistently found that women develop PTSD more frequently than men and that PTSD severity and chronicity tend to be greater in women [68]. In the present study, the PTSD group was predominantly female (65.2%); however, no statistically significant sex difference was observed between groups, and our findings regarding sex differences should be interpreted cautiously. This discrepancy may be related to the cross-sectional design and the relatively small sample size.

This study has several limitations. First, due to the cross-sectional design, causal relationships between sleep disturbances and PTSD cannot be inferred. It remains unclear whether sleep disturbances contribute to the development of PTSD, whether PTSD symptoms lead to sleep disruption, or whether the relationship is bidirectional. Prospective longitudinal studies are needed to clarify temporal sequencing and potential causal mechanisms.

In addition, the reliance on questionnaire-based assessments may introduce response bias, including recall bias and social desirability effects. A further methodological consideration concerns the use of different informants for sleep-related measures. While sleep quality (PSQI) was based on adolescent self-report, sleep problems (CSHQ) and chronotype (CCTQ) were parent-reported. Informant discrepancies between adolescents and parents are well documented in the assessment of internalizing symptoms and sleep-related behaviors. Adolescents may provide more accurate information regarding subjective sleep quality, whereas parents may be more reliable observers of observable nighttime behaviors [69]. These differences in informant source may have introduced measurement variability, particularly in the assessment of chronotype. Although PTSD diagnoses were established through face-to-face clinical interviews according to DSM-5 criteria, future studies incorporating multi-informant approaches and objective sleep measures (e.g., actigraphy or polysomnography) would strengthen methodological rigor.

Second, the single-center design and relatively modest sample size may limit generalizability. The sample consisted exclusively of earthquake-exposed adolescents aged 12–18 years, which may restrict the applicability of the findings to other trauma types such as interpersonal violence, abuse, or war-related exposure. Cultural and geographic factors may also influence sleep patterns and PTSD symptom expression. Therefore, multi-center and cross-cultural studies including diverse trauma populations are warranted.

Nevertheless, this study also has notable strengths. To our knowledge, this is one of the few studies examining the relationship between chronotype and PTSD in adolescents and combining clinician-based PTSD diagnosis with validated sleep measures. Furthermore, similarity between groups in key sociodemographic variables that could act as confounders enabled a clearer evaluation of the relationships among sleep quality, sleep problems, chronotype, and PTSD. Future studies with larger samples and broader age ranges are warranted.

## 5. Conclusions

Adolescents with PTSD following the Kahramanmaras Earthquake Sequence exhibited poorer sleep quality and more pronounced sleep problems compared with earthquake-exposed controls. Sleep quality impairment and clinically significant sleep problems were independently associated with PTSD status and correlated with symptom severity. Although evening chronotype was more frequent among adolescents with PTSD and related to greater symptom severity, it was not independently associated with PTSD in multivariable analyses. These findings indicate that the relationship between chronotype and PTSD may be partly explained by underlying sleep disturbances rather than a direct association. Systematic assessment of sleep and early interventions targeting sleep problems may therefore support recovery in adolescents exposed to large-scale traumatic events. Prospective longitudinal studies are required to clarify temporal relationships between sleep disturbances, chronotype, and PTSD in adolescence.

## Figures and Tables

**Table 1:** Comparison of sociodemographic and earthquake-related variables.

		PTSD Group		Control Group		F	*p* ^a^
Age (Mean ± SD)		15.30 ± 2.04		14.80 ± 1.87		2.154	0.069
		*n*	%	*n*	%	X^2^	*p* ^b^
Gender	Female	60	65.2	60	55.0	2.145	0.152
	Male	32	34.8	49	45.0		
Place of Residence	City Center	69	75.0	81	74.3	1.948	0.378
	District	13	14.1	21	19.3		
	Rural Area	10	10.9	7	6.4		
Monthly Household Income	Below Minimum Wage	8	8.7	11	10.2	0.291	0.864
	Minimum Wage	23	25.0	24	22.2		
	Above Minimum Wage	61	66.3	73	67.6		
Parental Psychiatric Disorder	No	83	90.2	103	94.5	1.322	0.289
	Yes	9	9.8	6	5.5		
Earthquake-related Damage to Home	No Damage	14	15.2	22	20.4	5.581	0.233
	Mild Damage	57	62.0	52	48.1		
	Moderate Damage	7	7.6	14	13.0		
	Severe Damage	13	14.1	20	18.5		
	Destroyed	1	1.1	0	0		
Bereavement Due to the Earthquake	No	85	92.4	102	94.4	0.345	0.579
	Yes	7	7.6	6	5.6		
Out-of-province Accommodation After the Earthquake	No	36	40.0	28	25.9	4.445	0.047 *
	Yes	54	60.0	80	74.1		

*p* ^a^ values are from the independent-samples *t*-test. *p* ^b^ values are from chi-square analysis. * *p* < 0.05.

**Table 2:** Comparison of CPTS-RI, PSQI, CSHQ, and CCTQ scores.

	PTSD Group (Mean ± SD)	Control Group (Mean ± SD)	F	*p*	Cohen’s d
CPTS-RI Score	51.88 ± 8.64	21.62 ± 10.27	5.719	<0.001 *	3.18
PSQI Score	8.76 ± 3.50	4.32 ± 2.72	6.592	<0.001 *	1.42
CSHQ Score	50.72 ± 9.92	42.50 ± 6.96	10.898	<0.001 *	0.97
CCTQ Score	33.35 ± 5.18	29.96 ± 5.39	0.411	<0.001 *	0.64

*p* values are from independent-samples *t*-tests. CPTS-RI: Children’s Posttraumatic Stress Reaction Index. PSQI: Pittsburgh Sleep Quality Index. CSHQ: Children’s Sleep Habits Questionnaire. CCTQ: Children’s Chronotype Questionnaire. * *p* < 0.001.

**Table 3:** Comparison of CPTS-RI-, CCTQ-, PSQI-, and CSHQ-related categorical variables.

		PTSD Group		Control Group		X^2^	*p*
		*n*	%	*n*	%		
Posttraumatic Stress Reaction Severity	No	0	0	23	21.1	201.000	<0.001 *
	Mild	0 ^b^	0	43 ^a^	39.4		
	Moderate	0 ^b^	0	43 ^a^	39.4		
	Severe	74 ^b^	80.4	0 ^a^	0		
	Very Severe	18 ^b^	19.6	0 ^a^	0		
Chronotype	Morning type	4 ^a^	4.3	13 ^a^	11.9	21.894	<0.001 *
	Intermediate type	32 ^b^	34.8	65 ^a^	59.6		
	Evening type	56 ^b^	60.9	31 ^a^	28.4		
Sleep Quality	Good	17	18.5	78	71.6	56.398	<0.001 *
	Poor	75	81.5	31	28.4		
Clinical Significance Based on CSHQ Score	Not Clinically Significant	15	16.3	51	46.8	21.023	<0.001 *
	Clinically Significant	77	83.7	58	53.2		
Sleep Talking	Rarely	62	67.4	81	74.3	1.430	0.489
	Sometimes	23	25	23	21.1		
	Usually	7	7.6	5	4.6		
Restlessness and Movement During Sleep	Rarely	46 ^b^	50	77 ^a^	70.6	15.505	<0.001 *
	Sometimes	26 ^a^	28.3	27 ^a^	24.8		
	Usually	20 ^b^	21.7	5 ^a^	4.6		
Teeth Grinding During Sleep	Rarely	75	81.5	91	83.5	0.372	0.830
	Sometimes	12	13	14	12.8		
	Usually	5	5.5	4	3.7		
Waking Up Frightened Due to a Scary Dream	Rarely	58 ^b^	63	88 ^a^	80.7	10.602	0.005 *
	Sometimes	22 ^a^	23.9	18 ^a^	16.5		
	Usually	12 ^b^	13	3 ^a^	2.8		

CSHQ: Children’s Sleep Habits Questionnaire. *p* values are from chi-square analysis. Superscripts ^a^ and ^b^ indicate statistically significant differences between the groups (*p* < 0.05). * *p* < 0.01.

**Table 4:** Correlation analysis of CPTS-RI, PSQI, CSHQ, and CCTQ scores.

	CPTS-RI Score	PSQI Score	CSHQ Score	CCTQ Score
CPTS-RI Score	1.000	0.642 *	0.500 *	0.275 *
PSQI Score	-	1.000	0.507 *	0.300 *
CSHQ Score	-	-	1.000	0.418 *
CCTQ Score	-	-	-	1.000

CPTS-RI: Children’s Posttraumatic Stress Reaction Index. PSQI: Pittsburgh Sleep Quality Index. CSHQ: Children’s Sleep Habits Questionnaire. CCTQ: Children’s Chronotype Questionnaire. * *p* < 0.001.

**Table 5:** Binary logistic regression analysis of out-of-province accommodation, PSQI, CSHQ, and CCTQ variables.

	β	OR	95% C.I.		*p*
			Lower	Upper	
Out-of-province Accommodation	−0.252	0.777	0.371	1.626	0.503
Sleep Quality based on PSQI Score 0: Sleep quality not impaired 1: Sleep quality impaired 0 → 1	2.238	9.378	4.578	19.214	<0.001 *
Clinical Significance based on CSHQ Score 0: Not clinically significant 1: Clinically significant 0 → 1	0.999	2.716	1.192	6.186	0.017 *
Chronotype based on CCTQ 0: Morning type 1: Intermediate type 0 → 1	0.189	1.208	0.218	6.710	0.829
Chronotype based on CCTQ 0: Morning type 2: Evening type 0 → 2	0.420	1.522	0.433	5.345	0.512
CCTQ Total Score	0.024	1.025	0.899	1.168	0.717

PSQI: Pittsburgh Sleep Quality Index. CSHQ: Children’s Sleep Habits Questionnaire. CCTQ: Children’s Chronotype Questionnaire. Nagelkerke R^2^ = 0.420. * *p* < 0.05.

## Data Availability

The data that support the findings of this study are available from the corresponding author (Y.E.D.) upon reasonable request.
